# Supplementary material for: Chaotic behavior, sensitivity analysis and Jacobian elliptic function solution of M-fractional paraxial wave with Kerr law nonlinearity
Source: PLoS One. 2025 Feb 21;20(2):e0314681. doi: 10.1371/journal.pone.0314681 (PMC11844848; doi:10.1371/journal.pone.0314681)
Supplement: S1 Data — (DOCX) [file pone.0314681.s001.docx]

***Simulation Data***

In this work, we use some simulation value of the free parameters.

| Simulation data | Name phenomena |
| --- | --- |
| $\theta=-0.1, \varepsilon_{3}=-1,z=1,\varepsilon_{2}=0.2,\tau=1,g_{1}=-0.5,h_{1}=-0.5,h_{2}=-0.01,\varepsilon_{1}=-1,n=1.5$. | Real portion (Fig. 7(a)-7(c)) and imaginary portion (Fig. 7(d)-7(f)) show the interaction between periodic lump wave and periodic wave and the absolute (Fig. 7(g)-7(i)) form of the solution represents the interaction of kink and periodic wave |
| $\theta=-0.1, \varepsilon_{3}=1,z=1,\varepsilon_{2}=-0.2,\tau=1,g_{1}=0.5,h_{1}=-3,h_{2}=-1.5,\varepsilon_{1}=1,n=1.5$ | The real portion (Fig. 8(a)-8(c)) and imaginary portion (Fig. 8(d)-8(f)) show the double periodic wave and the absolute (Fig. 8(g)-8(i)) form of the solution represents the interaction of soliton wave and kink wave |
| $\theta=0.1, \varepsilon_{3}=2,\varepsilon_{2}=0.1,\tau=-2,g_{1}=0.1,h_{1}=3,h_{2}=3,\varepsilon_{1}=1,n=1.5$. | The real portion (Fig. 9(a)-9(c)) and imaginary portion (Fig. 9(d)-9(f)) show the periodic breather wave and the absolute (Fig. 9(g)-9(i)) form of the solution represents the kinky-periodic wave |
| $\theta=0.1,z=1, \varepsilon_{3}=2,\varepsilon_{2}=0.1,\tau=0.5,g_{1}=0.1,h_{1}=-0.5,h_{2}=3,\varepsilon_{1}=1,n=1.5$ | The real portion (Fig. 10(a)-10(c)) and imaginary portion (Fig. 10(d)-10(f)) show the double periodic wave and the absolute (Fig. 10(g)-10(i)) form of the solution represents the interaction between soliton wave and kink wave |
| $\theta=-0.1, \varepsilon_{3}=-1,z=1,\varepsilon_{2}=0.2,\omega=1,g_{1}=-0.5,h_{1}=-0.5,h_{2}=-0.01,\varepsilon_{1}=-1,n=1.5$ | The real portion (Fig. 11(a)-11(c)) and imaginary portion (Fig. 11(d)-11(f)) show the periodic wave and the absolute (Fig. 11(g)-11(i)) form of the solution represents the kink shape wave. |
| $\theta=-0.1, \varepsilon_{3}=1,x=1,\varepsilon_{2}=1,\omega=0.1,g_{1}=2,h_{1}=1,h_{2}=2,\varepsilon_{1}=-0.1,n=1.5$ | The real portion (Fig. 12(a)-12(c)) and imaginary portion (Fig. 12(d)-12(f)) show the periodic wave with breather and the absolute (Fig. 12(g)-12(i)) form of the solution characterizes the interaction between anti-kink and soliton wave. |
