# Supplementary material for: Chaotic behavior, sensitivity analysis and Jacobian elliptic function solution of M-fractional paraxial wave with Kerr law nonlinearity
Source: PLoS One. 2025 Feb 21;20(2):e0314681. doi: 10.1371/journal.pone.0314681 (PMC11844848; doi:10.1371/journal.pone.0314681)
Supplement: Attachment — Submitted filename: Reviewer Response.docx [file pone.0314681.s002.docx]

**Reviewer Response**

**Reviewer #1:**

I have read this paper carefully. I see that the authors work very hard but unfortunately they have not found new results. My remarks on this paper are the following.

1) I believe that the authors have to give references on Eq. (1) where this equation was first derived.

**Response: The necessary correction is done in section 1 and we added the reference in [19].**

2) After equation (1) the authors wrote that the expression in the y direction but I cannot see any function which depends on y direction. I see the only function R(x,z,t).

**Response: Thanks. The necessary correction is done.**

3) The Section (2.2) is devoted to consideration Eq. (2), but Eq. (2) is nonlinear ordinary differential equation.

**Response: The necessary correction is done.**

4) Solution of Eq. (2) is well-known but the authors present a few of exact solutions.

**Response: We appreciate the reviewer's comment. While it is true that the general solution to equation (2) is well-known, our contribution lies in presenting a few exact solutions in specific forms that may not be widely recognized or easily derived. These solutions provide additional insight into the behavior of the governing model under conditions, and we believe they add value to the existing body of knowledge.**

5) The looked for of Equation (2) using formula (3) but they did not say what q_1, q_2, and p_1.The authors present different formulas for solution of Eq. (2) but these solutions are the same. More interesting Section 2 of paper. They present the consideration the chaotic nature.

**Response: The necessary correction is done in section 2.2 and 3.**

6) However the approach by authors is well known as well. See , for example, the book “Chaotic Transitions in deterministic and Stochastic Dynamical Systems” by Emil Simiu, Prinston University Press. Taking into account the remarks above unfortinately I do not recommend this paper for publication.

**Response: The reviewer’s mentioned paper described Melnikov's theory for chaotic dynamics. However, our paper uses a planar dynamical system to analyze the chaotic behavior of the governing model.**

**Reviewer #2: Review of the article:**

Chaotic behavior, sensitivity analysis and Jacobian elliptic function solution
of M-fractional paraxial wave with Kerr law nonlinearity.
Manuscript Number: PONE-D-24-28379

ESSENTIALS OF THIS ARTICLE:
In this paper the authors find several solutions of a fractional version of the 2D NLS (two-dimensional nonlinear Schrödinger) equation (which the authors call “paraxial wave equation”). In this article “x” is the evolution variable, and “t” and “z” are the “transversal” variables. The second-order time derivative has been replaced by an M-fractional derivative, which is a generalization of the conformable derivative.
Proposing a cleverly chosen similarity reduction, defined by the equations shown in (6), and the form of R(x,z,t) written above Eq. (6), the authors manage to reduce the fractional 2D NLS equation (1) to the ODE (ordinary differential equation) shown in (7). Then several particular solutions of this ODE are obtained by means of the EJEFE method.
Afterwards the authors study a perturbed form of Eq. (7) [shown in (17)], and obtain quasiperiodic and chaotic solutions of this perturbed equation.

OPINION OF THE ARTICLE:
The fractional 2D NLS equation studied in this article is an interesting equation, the solutions obtained are interesting, and the procedure to obtain these solutions is also interesting. The reduction of the fractional 2D NLS equation (1) to the ODE (7) is an excellent result (the most interesting result of the paper, from my point of view).
HOWEVER, the paper contains some errors and undefined symbols, which are probably the consequence of a hasty and careless writing. Moreover, some of the statements made in this article are, in my opinion, incorrect.

ISSUES THAT SHOULD BE CORRECTED:
ISSUE 1:
In the fourth line below Eq. (1) it is written “in the y and t directions”. This statement is wrong. It should say “in the t and z directions”.

**Response: The necessary correction is done.**

ISSUE 2:
In pag. 3 of the paper the authors mention References [34]-[43].
But the list of references presented at the end of the article only contains 32 references!!!

**Response: The necessary correction is done.**

ISSUE 3:
In the definition of the M-fractional derivative the meaning of the “t” with subscript kappa (which appears within the argument of the function “u”) is not explained. Moreover, in the right-hand-side (rhs) of the equation which defines this derivative, the parameter “M” does not appear. Please explain.

**Response: The necessary explanation is done in section 2.1**

ISSUE 4:
Below the definition of the M-derivative, in the section “Features”, in the equations (a), (b), (c) and (f), the meaning of the letters with the subscript kappa (le letters l, m, u and v) is not explained.

**Response: The necessary correction is done.**

ISSUE 5:
Two lines below Eq. (2) the authors say that:
“Balancing H’’ and H3, yields M=1”
but the authors have not said what is “M”. As I said in the Issue 3, the parameter M does not appear in the rhs of the equation which defines de M-derivative. So, it is incomprehensible how the equation M=1 is obtained.

**Response: We explain it in section 2.2 and section 3.**

ISSUE 6:
In the first line below Eq. (5) the authors mention the equations (6)-(8), but these equations have not been presented at this point of the article. Moreover, it is quite clear that in this line [below Eq. (5)], the authors are not referring to the Eqs. (6)-(8) which appear in Section 3.

**Response: Thanks. The necessary correction is done.**

ISSUE 7:
Two lines below Eq. (9) the equation M=1 appears again. As I said in Issue 5, we don´t know what is M, and therefore the origin of the equation M=1 is incomprehensible.
**Response: we explain it in section 2.2.**

ISSUE 8:
Two lines above Eq. (17) the authors mention a “superficial component”. This term is a misleading, as it is not related to any “surface” at all. Moreover, as far as I could see, this term is never mentioned in Ref. [31].
**Response: We modified it.**

ISSUE 9:
In the line above Eq. (17) the authors mention the “system Eq. (30)”. But the article contains 17 equations, consequently the mention of Eq. (30) is obviously incorrect.

**Response: We modified it.**

ISSUE 10:
The numbering of the sections of this article is an absolute disaster, revealing an extremely careless work.
In page 3 the Sec. 2 begins.
In page 5, Sec. 3 begins.
Then, in page 6, another “Section 2” begins.
Then, in page 8, the Section 8 begins!.
And in page 9, the Section 5 begins!
Finally, in page 14, the Section 6 closes the paper!

**Response: We modified it.**

ISSUE 11:
In page 6, in the Section entitled “Chaotic nature”, the authors modify their Eq. (7), introducing an oscillatory perturbation in time [the last term in the second equation shown in (17)]. And then they show that the solutions of this new ODE [Eq. (17)] exhibit a chaotic behavior. But the chaotic behavior of the solutions of Eq. (17) does not imply that Eq. (7) [or Eq. (1)] is a chaotic system.
The chaotic nature of a system (i.e., of an equation), is proved by perturbing THE INITIAL CONDITIONS USED, and NOT by perturbing the equation itself. Even though some authors may consider that an equation is “chaotic” if the solutions exhibit a huge change when the equation is perturbed, THIS IS NOT THE USUAL DEFINITION OF CHAOS. At least, it is not the definition of “chaos” used by Edward Lorenz (who was one of the founders of the theory of chaos).
Consequently, I consider that the authors must explain clearly that the chaotic solutions of Eq. (17) do not imply that the system described by Eq. (7) [or Eq. (1)] is chaotic.

**Response: We include the following paragraph in the “Chaotic nature” section:**

It is important to note that the chaotic behavior observed in the solutions to Eq. (17) arises due to the introduction of an oscillatory perturbation to the system. This does not necessarily imply that the original system described by Eq. (7) [or Eq. (1)] is chaotic in the traditional sense of chaos theory, where chaos is typically characterized by sensitivity to initial conditions rather than sensitivity to perturbations in the system itself. This distinction aligns with the definition of chaos introduced by Edward N. Lorenz and is crucial for accurately characterizing the system's dynamics [37].

**[37] Lorenz EN. The Essence of Chaos. University of Washington Press. 1993; 181–206.**

This clarification should help address the reviewer's concern while maintaining the integrity of your analysis.

**Reviewer #3:**

The authors investigated various types of wave solutions in the M-fractional paraxial wave equation with Kerr law nonlinearity, including periodic waves, lumpperiodic waves, periodic breather waves, kink-bell waves, kinky-periodic waves, anti-kinky-periodic waves, double-periodic waves. In addition, the chaotic phenomena are also studied in this work. The wave equations with M-fractional derivative attracted many attentions in recent years and the results presented in this work are interesting. However, some issues should be fixed, and my comments are as follows:

1. The physical scene (settings) for M-fractional derivative may be a good supplement to this work.

**Response: Thanks for your response.**

2. I am wondering if such M-fractional derivative can be expressed by other easier forms that can be solved numerically.

**Response: Thanks**

3. Where is the Eq. (30) the authors pointed out before Eq. (17) ?

**Response: we modified it.**

4. I assume the references of [34] to [42] in the Introduction should be wrong. The authors should check it.
**Response: we modified it.**
5. Some review should be added for the better understanding of fractional derivative.

**Response: We cited some related article in [16, 17, 18].**

6. PLOS authors have the option to publish the peer review history of their article ([what does this mean?](https://journals.plos.org/plosone/s/editorial-and-peer-review-process#loc-peer-review-history)). If published, this will include your full peer review and any attached files.


If you choose “no”, your identity will remain anonymous but your review may still be made public.
