# Supplementary material for: Chaotic behavior, sensitivity analysis and Jacobian elliptic function solution of M-fractional paraxial wave with Kerr law nonlinearity
Source: PLoS One. 2025 Feb 21;20(2):e0314681. doi: 10.1371/journal.pone.0314681 (PMC11844848; doi:10.1371/journal.pone.0314681)
Supplement: Attachment — Submitted filename: Reviewer Response.docx [file pone.0314681.s003.docx]

Reviewer #2: Review of the revised version of the article:
Chaotic behavior, sensitivity analysis and Jacobian elliptic function solution
of M-fractional paraxial wave with Kerr law nonlinearity.
Manuscript Number: PONE-D-24-28379R1

In the revised version of this article the authors have taken into account the observations mentioned in my previous review. However, two of the issues mentioned in that review have not been answered satisfactorily: Issues 3 and 4.
In page 4 the authors added a new paragraph entitled “Features”, aimed to clarify the Issues 3 and 4. However, the “explanations” contained in this paragraph are completely unsatisfactory. The statement:
“M indicates the truncation point or order”
is absolutely ambiguous, and it does noy clarify how the parameter M enters in the calculation of the limit which appears in the rhs (right hand side) of the equation which defines the M-derivative. Additionally, the statement:
“kappa represents a scaling factor or a characteristic constant associated with the operator”
is also imprecise and ambiguous, as the letter “kappa” enters in the rhs of the definition of the M-derivative as the name of a subindex, and it does not occupy the position of a numerical factor or a constant. Therefore, it is necessary to explain with absolute clarity how the symbol t_kappa depends on kappa.
And, in a similar way, the authors do not explain how the parameters l_kappa, m_kappa, u_kappa and v_kappa, which appear in the equations (a), (b) and (c) presented in page 4, depend on kappa. Therefore, it is necessary to explain with absolute clarity how these four symbols depend on kappa.

**Ans.: In this operator**${}_{\boldsymbol{\kappa}}{\boldsymbol{D}_{\boldsymbol{M,t}}^{\boldsymbol{\sigma,\phi}}}$**,** $\boldsymbol{\kappa}$ **is real constant, known as a scaling factor or a characteristic constant associated with the operator. It is related to the operator through the truncated Mittag-Leffler function with one parameter, as discussed in [40]**

${}_{\boldsymbol{\kappa}}{\boldsymbol{E}_{\boldsymbol{\phi}}}\left( \boldsymbol{t} \right)\boldsymbol{=}\sum_{\boldsymbol{n=0}}^{\boldsymbol{\kappa}} \frac{\boldsymbol{t}^{\boldsymbol{n}}}{\boldsymbol{\Gamma}\left( \boldsymbol{\phi n + 1} \right)}$**.**

**In the feature,** $\boldsymbol{u}\boldsymbol{=u}\left( \boldsymbol{t} \right)\boldsymbol{,v}\boldsymbol{=v(t)}$ **are the function of time** $\boldsymbol{t}$**. We justify the properties of truncated M-fractional operator.**

**The parameter 𝑀 is used to denote that the function to be derived involves a Mittag-Leffler function with one parameter. Further discussion can be found in [41].**

${{}_{\kappa}{D_{M,t}^{\sigma,\phi}}}u\left( t \right)=\lim_{\epsilon\to0} \frac{u\left( t_{\mathcal{K}}E_{\phi}\left( \epsilon t^{-\sigma} \right) \right)-u\left( t \right)}{\epsilon}, t>0,\phi>0$.

Here, ${}_{\boldsymbol{\kappa}}{\boldsymbol{E}_{\boldsymbol{\phi}}}\left( \boldsymbol{.} \right)$is a truncated Mittag-Leffler function of one parameter. In left side M covert into ${}_{\boldsymbol{\kappa}}{\boldsymbol{E}_{\boldsymbol{\phi}}}\left( \boldsymbol{.} \right)$ **In right side. (See [41] (definition 2.1, 2.2))**

Recommendation:
As the essential novelty of the equation studied in this article is the use of the M-derivative, the definition and the properties of this operator must be absolutely clear in the article. Therefore, in my opinion, this article should not be accepted for publication in PLOS ONE until these issues are presented with absolute clarity.

Reviewer #3: The authors have replied all my comments. This manuscript looks much better now. I have no further comments on this work.

Thanks for your recommends.
